# Wild cucumber invasiveness: impact of seasonal changes on biometric seed traits and dispersal strategy

**DOI:** 10.1093/jxb/eraf526

**Published:** 2025-12-04

**Authors:** Alicja Dołkin-Lewko, Paweł Baj, Aleksandra Giedrowicz, Jakub Jaroszewicz, Urszula Zajączkowska

**Affiliations:** Department of Forest Botany, Institute of Forest Sciences, Warsaw University of Life Sciences—SGGW, Warsaw 02-776, Poland; Faculty of Power and Aeronautical Engineering, Warsaw University of Technology, Warsaw 00-661, Poland; Warsaw University of Life Sciences – SGGW, Warsaw 02-776, Poland; Faculty of Materials Science and Engineering, Warsaw University of Technology, Warsaw 00-661, Poland; Department of Forest Botany, Institute of Forest Sciences, Warsaw University of Life Sciences—SGGW, Warsaw 02-776, Poland; University of Cambridge, UK

**Keywords:** Bet-hedging, digital image correlation, hydrochory, invasion, seed buoyancy, seed transport, temporal dispersal

## Abstract

Wild cucumber (*Echinocystis lobata*) is an invasive annual vine rapidly spreading across Central European riparian zones. Its success is linked to seed dispersal, with hydrochory expected to play a major role, but the specific mechanisms are not fully understood. This study examines whether *E. lobata* uses a dual dispersal strategy by seed morphological differences and hydrodynamic behavior, supporting both short-distance and long-distance dispersal. Seeds were collected in autumn and early spring, and analysed for morphology, buoyancy, and hydrodynamic properties. Over 150 seeds underwent biometric measurements: digital image correlation for swelling deformation, micro-computed tomography for internal structure, and hydrodynamic properties. Approximately 23% of seeds were buoyant, with buoyant ones present in both autumn and spring seed groups. Buoyant seeds had larger surface areas and internal air cavities, contributing to lower drag and enhanced passive transport in water. Using digital image correlation and micro-computed tomography imaging, we visualized seed coat swelling patterns and quantified internal structures. Hydrodynamic experiments revealed that buoyant seeds experienced lower drag and slower acceleration. Under oscillatory flow, buoyant seeds exhibited passive propulsion at 1 Hz, indicating a resonance effect facilitating dispersal during fluctuating water flow. These findings support a dual dispersal strategy: temporal seed release and buoyancy enable both local recruitment and long-distance dispersal, likely contributing to invasive success in riparian habitats.

## Teaser

Wild cucumber shows seasonally induced differences in seed structure and morphology, resulting in variable buoyancy and a dual dispersal strategy that may enhance its invasiveness across dynamic riparian environments.

## Introduction

Rush habitats, wetlands, and river valleys are particularly vulnerable to invasion by alien species because they create favorable conditions for their colonization (DeFerrari and Naiman, 1994). Anthropogenic effects, such as habitat modification and proximity to urban areas, further enhance invasion risk (Knapp *et al*., 2010; Dyderski and Jagodziński, 2016). Climate change may additionally influence the spread of invasive alien species by altering habitat conditions and disturbance dynamics (Hellmann *et al*., 2008). Uncontrolled expansion of invasive species, especially in areas with strong anthropogenic pressure, may result in unfavorable natural succession and, consequently, destabilization of the ecological system in a given area (Fortuna-Antoszkiewicz *et al*., 2018). Alien species can completely disrupt the network of ecological connections in current ecosystems (Faliński, 2004) or create monotypes, which leads to a change in the structure of habitats and a decrease in biodiversity (Zedler and Kercher, 2004). The reduction in the number of native species due to the arrival of alien species has ecological consequences, but also poses a threat to the dominant landscape (Hejda *et al*., 2009).

Similar phenomena are observed in forest areas, especially in riparian and alder communities, where plants from the vine group play an important role. An example is the wild cucumber (*Echinocystis lobata* (F. Michx.) Torr. & A. Gray), which has significantly increased its area of occurrence over the last few decades (Zając *et al*., 2011). Native to North America, this species was introduced to Europe in the 19th century as an ornamental plant and has since become invasive in Central and Eastern Europe, including Poland, Lithuania, Latvia, and the Czech Republic, and is now spreading to other parts of Europe such as Germany, Austria, and Hungary (Bagi and Böszörményi, 2008; Tokarska-Guzik *et al*., 2015; Jocienė *et al*., 2023). It thrives in temperate climates and tolerates periodic flooding, benefiting from warm growing seasons and nutrient-rich alluvial soils (Kostrakiewicz-Gierałt *et al*., 2022). For this reason, it has been included by the Polish Ministery of the Environment on the list of species threatening native flora or natural habitats, as well as on the list of 100 most invasive species of plants, animals, and fungi in Europe (Vilà *et al*., 2009); therefore, appropriate management and planning of conservation measures are often crucial in preserving particularly valuable natural areas (Vicente *et al*., 2013).

Wild cucumber prefers rushes, scrubs, and river forest habitats, and is also present in anthropogenic habitats such as homesteads and roadsides. Despite its wide range, it poses the greatest threat to native biodiversity in riverside herbaceous areas and the edges of riparian forests, a significant part of which belong to Natura 2000 habitats. Such habitats include hydrophilous tall herb fringe communities, rivers with muddy banks, and riparian forests (Priede, 2008; Kołaczkowska, 2010). The range of wild cucumber increases along river banks, due to seed transfer by running water, facilitating rapid colonization of subsequent sections of the shoreline (Bagi and Böszörményi, 2008). In the context of dispersal ecology, these processes can be classified both as short-distance dispersal, supporting local population persistence, and long-distance dispersal, enabling colonization of new areas (Nathan *et al*., 2008; Marco *et al*., 2011). Wild cucumber is a very light-demanding species that reduces light availability for native plants, inhibiting their growth and contributing to a decline in biodiversity. As it grows, it climbs onto surrounding herbaceous plants, shrubs, and trees using tendrils. It is characterized by very rapid growth during the vegetative period and reproduces exclusively by seeds with a long lifespan (Jocienė *et al*., 2023).

Despite the growing threat that wild cucumber poses to native ecosystems, there is still a lack of research on the mechanisms of its spread, especially in the context of seed transfer by running water. An important goal of research on this species is to determine the temporal and spatial patterns in the dispersal of its seeds. This research focused on seeds collected during two distinct periods—autumn and early spring—to analyse differences in their biometric features. Their buoyancy and ability to move in water were also examined. Furthermore, we analysed internal structural changes during swelling, including seed coat deformation. Such a comprehensive approach will allow for a better understanding of the biology of wild cucumber spread and may contribute to the development of effective control strategies.

In this study, we aim to answer the question of whether wild cucumber seeds exhibit both temporal and spatial variation in dispersal, allowing them to establish stable populations while colonizing new areas.

We hypothesize that (i) seeds collected in autumn and after winter (spring) differ in morphology and buoyancy, reflecting different fates during the dispersal season, and (ii) variation in seed traits affects their floating ability and hydrodynamic behavior, which may determine the potential distance of water-mediated dispersal.

## Materials and methods

### Study species

Wild cucumber (*Echinocystis lobata* (F. Michx.) Torr. & A. Gray) is an annual, fast-growing climbing vine from the *Cucurbitaceae* family. It reproduces exclusively by seeds, which mature in autumn and are released gradually: most fall soon after the fruit opens, while some are shed later as the capsule dries (Klotz, 2009). Fruits are spiny, 2.5–5 cm long capsules usually containing four flattened seeds (Silvertown, 1985; Dylewski *et al*., 2018). Seeds are brown, about 1.5–2.0 cm long and 0.5–1 cm wide, with a hard, water-impermeable coat and long viability (Klotz, 2009). Fresh seeds are dormant and require cold stratification to germinate (Choate, 1940; Bagi and Böszörményi, 2008; Grašič *et al*., 2019). Reported thousand-seed weight is 256.8–293.0 g, with specific gravity around 1.08–1.10 g cm⁻³ (Bagi and Böszörményi, 2008).

### Research material

The seeds of wild cucumber used in the experiment were collected from a natural site near the village of Gassy, located near Warsaw, Masovian Voivodeship, Poland (52.078872, 21.207374) in two periods, autumn (September and October 2020) and early spring (March in 2021), and from a second location on the river bank in Konstancin-Jeziorna near Warsaw, Masovian Voivodeship, Poland (52.101174, 21.127691) in autumn (September 2024). The sites were located in the floodplain areas of the Vistula River and were similar to the Populetum albae Br.-Bl. 1931 habitat. Both sites are located within the same river valley and are ∼6–7 km apart, with comparable riparian vegetation and hydrological conditions. Seeds collected in 2020/2021 were measured directly after natural drying, and seeds collected in 2024 were analysed fresh to avoid any potential effects of long-term storage.

### Biometric characteristic and buoyancy of seeds

To examine the biometric characteristics of seeds, three groups were analysed: 50 dry seeds collected in autumn 2020, 50 dry seeds collected in spring 2021 (which had remained inside fruit capsules over the winter), and 50 freshly collected (hydrated) seeds from autumn 2024. In natural conditions, wild cucumber seeds are released from the fruit in autumn while still moist, whereas those that remain enclosed in the capsules until spring gradually dry out over winter. Including dry autumn seeds enabled a direct comparison with naturally dried spring seeds. Each seed was weighed on a Kern ADB 200-4 scale (Kern & Sohn, Balingen, Germany) to four decimal places and scanned using a high-resolution scanner (Epson Perfection V700, Epson, Suwa, Japan). The scans were used to measure biometric characteristics (length, width, circularity, area, perimeter). The parameters were measured using the ImageJ program (Schneider *et al*., 2012). Circularity was calculated as:

$$\mathrm{Circularity}=\frac{4\pi Area}{{\mathrm{Perimeter}}^{2}}$$

Additionally, the buoyancy of each seed was checked. For this purpose, each seed was placed in a container with water. The seeds that were floating were removed and placed back in a vial with water, where their fall time was measured for 24 h. To exclude the possibility that the buoyant seeds were damaged or empty and unable to germinate, an experiment was carried out to study the germination ability of both buoyant (floating) and non-buoyant (sinking) seeds collected in 2020/21. For this purpose, 10 buoyant seeds and 10 non-buoyant seeds were selected from both periods. These seeds were placed on a plate with moist lignin and then placed in a refrigerator for 14 d. After this time, the seeds were placed on wet lignin, under controlled lighting (HPS Phytolite 600 W lamp, photon flux 1045 µmol m^−2^ s^−1^, luminous flux 100 klm) and temperature (21 °C). To verify the viability of seeds from both groups, we performed a 2,3,5-triphenyltetrazolium chloride (TTC) test on a larger sample, following the method of Ciacka *et al*. (2022) with minor modifications. A total of 50 buoyant and 50 non-buoyant seeds, freshly collected in autumn, were used. As a control, one seed from each group was boiled in water for 10 min before staining to confirm that dead tissue would not become colored. The isolated embryos were briefly rinsed twice in distilled water and then placed in a solution of 2% (w/v) TTC (Sigma-Aldrich, St Louis, MO, USA) prepared in 0.9% (w/v) sodium chloride (Sigma-Aldrich) with the addition of 2 mM dimethyl sulfoxide (Sigma-Aldrich). Staining was performed for 90 min at room temperature in darkness. After incubation, embryos were washed with distilled water and photographed using a digital microscope (Tagarno FHD Trend). Red coloration of the embryo axis was interpreted as dehydrogenase activity and therefore viability. The boiled negative controls remained unstained, confirming the validity of the test. The photographic documentation of both the germination and TTC tests is provided in Supplementary Figs S1–S3.

Then, using STATISTICA 13.1 software (TIBCO Software Inc., 2017), the measured parameters were compared for seeds from different harvest periods and conditions (dry/fresh). The same seeds were also categorized based on their ability to float. For this purpose, statistical tests were performed at a significance level of 5%. The relationships between seed biometric traits and the harvest period and conditions were assessed using the non-parametric Kruskal–Wallis analysis of variance, and homogeneous groupings were identified with a multiple comparison test. The relationships between seed buoyancy and biometric traits were analysed using the non-parametric Mann–Whitney *U*-test.

### Changes in seed structure during swelling

Tissue deformations during seed swelling were studied using the digital image correlation (DIC) method based on two cameras in a stereoscopic system connected to a stereoscopic microscope. This technique allowed for 3D deformation and strain measurements of specimens. Each camera provided a 2D view, and the software, using a correlation algorithm, created a 3D image by combining both camera views from different angles (Rusin and Kopernik, 2016). Deformation measurements of the seed coat of wild cucumber were performed using the Dantec Q-400 system, based on the Istra 4D software module with 5 MPix optics, connected to a Leica M125 stereoscopic microscope (Leica, Wetzlar, Germany). To prepare the sample for research and create a pattern on the surface, white dots were applied to the seed coat using acrylic paint and a small brush. The seed was placed on a watch glass covered with moist lignin, the ends of which remained in contact with water throughout the observation period to prevent the material from drying out. The changes in the pattern formed on the seed coat due to deformation and swelling were recorded by cameras. These images were automatically analysed. As a result, a dataset was generated, containing the initial contour of the object at the beginning of the measurement and the three-dimensional displacement vector of each point of the object due to its deformation. In this experiment, deformation patterns were analysed in 10 randomly selected dry seeds. For the purpose of visualizing representative swelling patterns, one example was selected and presented for each identified type of response.

### Analysis of the internal structure of seeds

Micro-computed tomography (micro-CT) analysis was conducted to examine the internal structure of wild cucumber seeds, focusing on differences in tissue organization, air cavity distribution, and seed coat thickness between buoyant and non-buoyant seeds. Three seeds from each group, freshly collected in autumn 2024, were selected, and their biometric characteristics, including length, width, and mass, were measured prior to scanning. Micro-CT scanning was performed using a MICRO XCT-400 system (Xradia, Zeiss, Oberkochen, Germany) at a voltage of 40 kV and a current of 250 μA. For each sample, 1200 projection images were acquired with an exposure time of 4 s per image using an LFOV camera. The scans achieved an isotropic voxel size of 19.8 μm, enabling detailed structural analysis. The reconstruction of the scanned volume was carried out using the instrument’s proprietary software. The reconstructed datasets were then exported to Avizo Fire (FEI Visualization Sciences Group) for advanced 3D image analysis, allowing for detailed visualization and quantitative evaluation of the sample’s microstructure. The analysis focused on segmenting and measuring key seed components, including the seed coat to determine its thickness and structural integrity, internal air spaces to assess their role in buoyancy, and the internal seed tissues comprising the cotyledons and embryo. Data from three independent measurements per seed type were compiled into a summary table.

### Hydrodynamic forces of seeds

Hydrodynamic forces acting on the seeds were estimated indirectly by analysing their trajectories while being advected by water. According to Newton’s second law of motion, the effective force was inferred from the seed’s acceleration, computed as the second derivative of its recorded trajectory. Two distinct flow scenarios were considered to assess seed motion under different hydrodynamic conditions (referred to as channel and vessel experiments hereafter). In the first scenario (channel experiment), seeds initially at rest were introduced into a steady water stream. In each experiment, 20 buoyant and 20 non-buoyant dry seeds were used. Each seed was tethered using a nylon fishing line (0.06 mm in diameter), glued to the seed at one end and fixed at the other, which was then suddenly released. The seed’s motion was tracked until it reached the free-stream velocity *U_∞_*=0.24 m s^−1^. For floating seeds, the tethering point was selected to keep the fishing line horizontal, minimizing vertical forces. The line was sufficiently flexible and assumed to transmit only tensile forces. The attachment point was chosen to preserve the natural orientation of the seed. Measurements were carried out in a closed-loop water tunnel with a cross-sectional area of 260 mm×35 mm. Trajectories were recorded with a 70 Hz camera (Imager E-Lite, LaVision) fitted with a 70 mm lens (Sigma 24–70/2.8 Ex Dg Macro), with calibration performed to convert pixels to physical units. In the second scenario (vessel experiment), seeds were placed at the center of a shallow vessel (165 mm×80 mm, 15 mm depth), mounted on a linear translation table oscillating sinusoidally along its short axis (i.e. *x*-axis). The table’s motion followed:

$$x_{\mathrm{table}}(t)=A\sin(2\pi ft),u_{\mathrm{table}}(t)=2\pi f\times A\cos(2\pi ft),a_{\mathrm{table}}(t)=-4\pi^{2}f^{2}\times A\sin(2\pi ft),$$

where *A* is the oscillation amplitude and *f* the frequency. Frequencies ranged from 0.5 to 3 Hz in 0.5 Hz steps. Table 1 summarizes the parameters of table motion. The surface flow was visualized using cork dust particles (diameter of 0.2 mm), assumed to follow the surface flow with negligible slip. Trajectories were recorded over at least 25 oscillation periods with a minimum of 20 frames per period. Recordings affected by wall-seed adhesion were discarded and repeated.

**Table 1.** Parameters of motion of the linear translation table

	Frequency (Hz)					
	0.5	1.0	1.5	2.0	2.5	3.0
max *x*_table_=*A* (mm)	49.2	37.1	11.3	3.1	1.9	6.8
max *u*_table_=*2*π*fA* (mm s^−1^)	155	233	107	39	30	128
max *a*_table_=*4*π*^2^f ^2^A* (mm s^−2^)	485	1464	1005	488	473	2404

A, oscillation amplitude; *f*, frequency.

The drag coefficient *C*_D_ was calculated using:

$$C_{D}=\frac{2a\ell \;}{\;u^{2}}\frac{m}{\rho_{w}\ell^{3}}$$

where *a* and *u* are the seed’s acceleration and velocity, *m* is the seed mass, *ℓ* is the seed characteristic length (evaluated as square root of the platform area), and *ρ*_w_ is the water density. The Reynolds number (Re) was defined as:

Re=(U∞−u)ℓ/v

where *ν* is the kinematic viscosity of water.

To analyse vessel experiments, trajectories were phase-averaged with respect to the table oscillation phase ϕ=2πft. The phase-averaged velocity $\hat{u}=[{\hat{u}}_{x},{\hat{u}}_{y}]$ was obtained via differentiation of the averaged trajectories and expanded as a Fourier series:

$$\hat{u}=\overset{\infty }{\underset{k=0}{\sum }}\;{\hat{u}}_{k}\cos\;(k\phi +\theta_{k})$$

where ${\hat{u}}_{k}=[{\hat{u}}_{x,k}$,${\hat{u}}_{y,k}]$ are amplitudes of Fourier coefficients and $\theta_{k}=[\theta_{x,k},\theta_{y,k}]$ their corresponding phase lags.

## Results

### Biometric characteristic and buoyancy of seeds

The biometric analysis of wild cucumber seeds collected in different seasons (autumn and spring) and at different moisture states (dry and fresh) revealed significant variations in seed morphology and buoyancy (Tables 2, 3). Seeds collected in autumn were generally smaller compared with seeds collected in spring (both dry). Similarly, autumn seeds exhibited a smaller width and mass, while spring seeds were wider and heavier. Fresh autumn seeds did not differ statistically from spring seeds. The only differences were visible in circularity, where fresh autumn seeds resembled those in a dry state, and width, where they differed significantly from the other groups. Autumn seeds were rounder, and spring seeds were elongated. Seed viability and germination potential of both buoyant and non-buoyant seeds were verified by a preliminary germination test and a TTC test, providing the basis to also analyse their biometric traits separately (Supplementary Figs S1–S3). A total of 150 wild cucumber seeds were tested for buoyancy, including seeds collected in both autumn and spring, in both dry and fresh states. These were the same seeds used in the biometric analyses. Of all the seeds tested, 34 (approximately 23%) remained afloat, while 116 sank. The ability to float varied between seed groups, with the highest proportion observed among freshly collected autumn seeds (32%, where only 10% of dry autumn seeds and 26% of dry spring seeds were buoyant). Buoyant seeds differed significantly from non-buoyant ones in nearly all morphological parameters, such as length, width, and surface area, except for mass, which showed no significant difference.

**Table 2.** Comparison of seed traits across harvest seasons and moisture states (*n*=50 per group)

Seed group	Homogenous groups	Mean	Median	Minimum	Maximum	Standard deviation	Coefficient of variation
Mass (g)							
Autumn (dry)	A	0.27	0.26	0.21	0.33	0.03	11.17
Spring (dry)	B	0.35	0.36	0.27	0.43	0.04	9.88
Autumn (fresh)	B	0.34	0.34	0.21	0.49	0.06	16.80
Length (mm)							
Autumn (dry)	A	15.83	15.89	13.77	18.46	1.14	7.21
Spring (dry)	B	18.65	18.64	16.66	20.99	0.84	4.52
Autumn (fresh)	B	18.92	18.61	16.20	22.20	1.56	8.25
Width (mm)							
Autumn (dry)	A	8.43	8.54	6.89	9.30	0.57	6.78
Spring (dry)	B	9.18	9.18	7.81	10.32	0.65	7.09
Autumn (fresh)	C	9.73	9.85	8.14	10.85	0.72	7.39
Surface area (mm^2^)							
Autumn (dry)	A	98.93	98.67	75.49	122.34	10.25	10.36
Spring (dry)	B	127.41	125.51	106.29	157.69	11.44	8.98
Autumn (fresh)	B	137.06	134.00	102.90	170.97	18.67	13.62
Perimeter (mm)							
Autumn (dry)	A	39.40	39.34	35.13	44.23	2.18	5.54
Spring (dry)	B	45.28	44.94	41.56	50.15	1.90	4.20
Autumn (fresh)	B	46.23	45.70	41.07	53.24	3.42	7.40
Circularity							
Autumn (dry)	A	0.80	0.80	0.73	0.88	0.03	4.30
Spring (dry)	B	0.78	0.78	0.73	0.84	0.03	3.62
Autumn (fresh)	A	0.80	0.81	0.74	0.86	0.03	3.95

Statistical differences were evaluated using the Kruskal–Wallis test; *P*<0.05.

**Table 3.** Comparison of seed traits between buoyant and non-buoyant seeds (*n*=34 for buoyant seeds, *n*=116 for non-buoyant seeds)

Seed group	Homogenous groups	Mean	Median	Minimum	Maximum	Standard deviation	Coefficient of variation
Mass (g)							
Non-buoyant	A	0.32	0.32	0.21	0.49	0.06	17.71
Buoyant	A	0.32	0.33	0.21	0.41	0.06	18.19
Length (mm)							
Non-buoyant	A	17.41	17.71	13.77	21.87	1.68	9.63
Buoyant	B	19.11	19.03	15.54	22.20	1.84	9.63
Width (mm)							
Non-buoyant	A	9.01	9.01	6.89	10.85	0.79	8.74
Buoyant	B	9.49	9.75	7.28	10.82	0.90	9.53
Surface area (mm^2^)							
Non-buoyant	A	117.19	117.01	75.49	169.98	19.18	16.36
Buoyant	B	134.59	136.36	87.73	170.97	23.16	17.21
Perimeter (mm)							
Non-buoyant	A	42.84	43.38	35.13	51.80	3.56	8.31
Buoyant	B	46.34	46.82	38.47	53.24	4.17	9.00
Circularity							
Non-buoyant	A	0.80	0.80	0.73	0.88	0.03	4.14
Buoyant	B	0.78	0.78	0.73	0.85	0.03	3.93

Statistical differences were evaluated using the Mann–Whitney *U*-test; *P*<0.05.

### Changes in seed structure during swelling

**Fig. 1.**
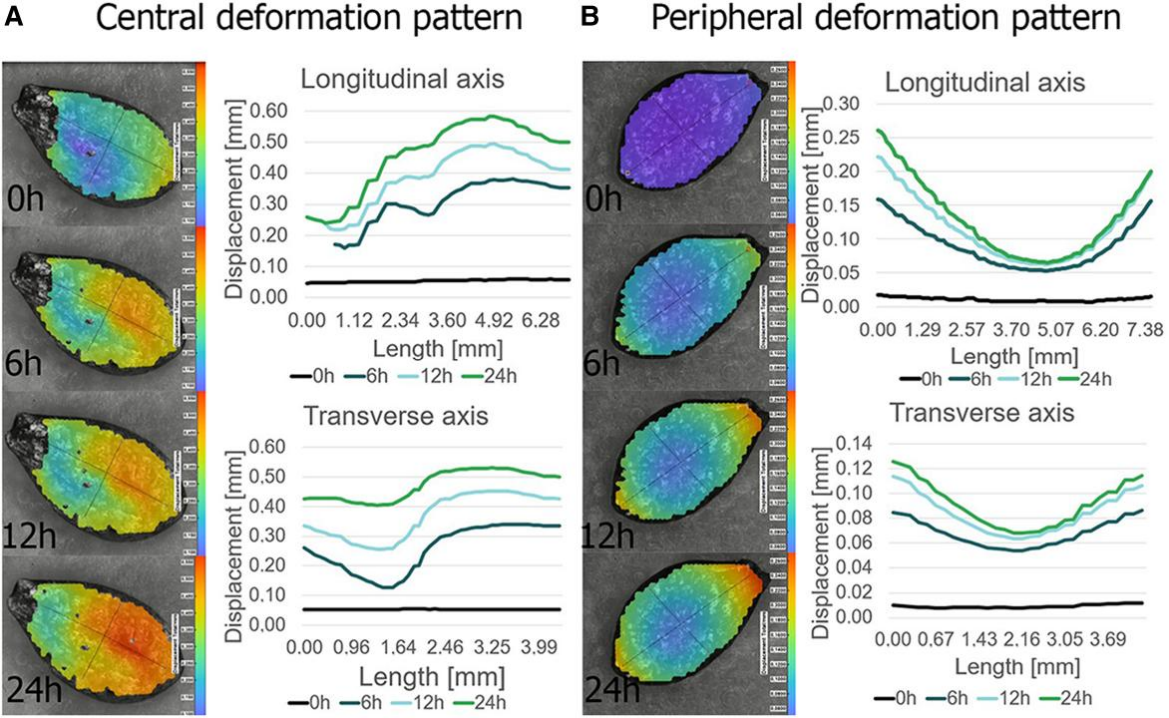
Deformation patterns in wild cucumber seeds during swelling, visualized using digital image correlation (DIC). (A) Central deformation pattern: deformation concentrated in the middle part of the seed. (B) Peripheral deformation pattern: deformation observed at the outer ends of the seed. For each case, four DIC images illustrate deformation distribution at 0 h (start), 6, 12, and 24 h of swelling. Color-coded deformation maps are accompanied by line plots showing deformation profiles along the longitudinal (seed length) and transverse (seed width) axes, derived from intersecting line sections marked on the seed surface.

DIC analysis of seed coat deformation during swelling revealed progressive structural changes in response to moisture absorption (Fig. 1; Videos 1, 2). The initial phase of swelling was characterized by a gradual increase in seed coat expansion, particularly in regions with thinner outer layers. Over time, local deformations indicated differential water uptake across seed tissues, with some areas absorbing moisture faster than others. Swelling patterns of wild cucumber seeds followed two predominant structural responses. In the first pattern (Fig. 1A; Video 1), swelling was primarily concentrated in the central region of the seed, where the cotyledons are located, leading to a noticeable upward deformation. In the second pattern (Fig. 1B; Video 2), swelling was most evident at the outer ends of the seed.

### Analysis of the internal structure of seeds

**Fig. 2.**
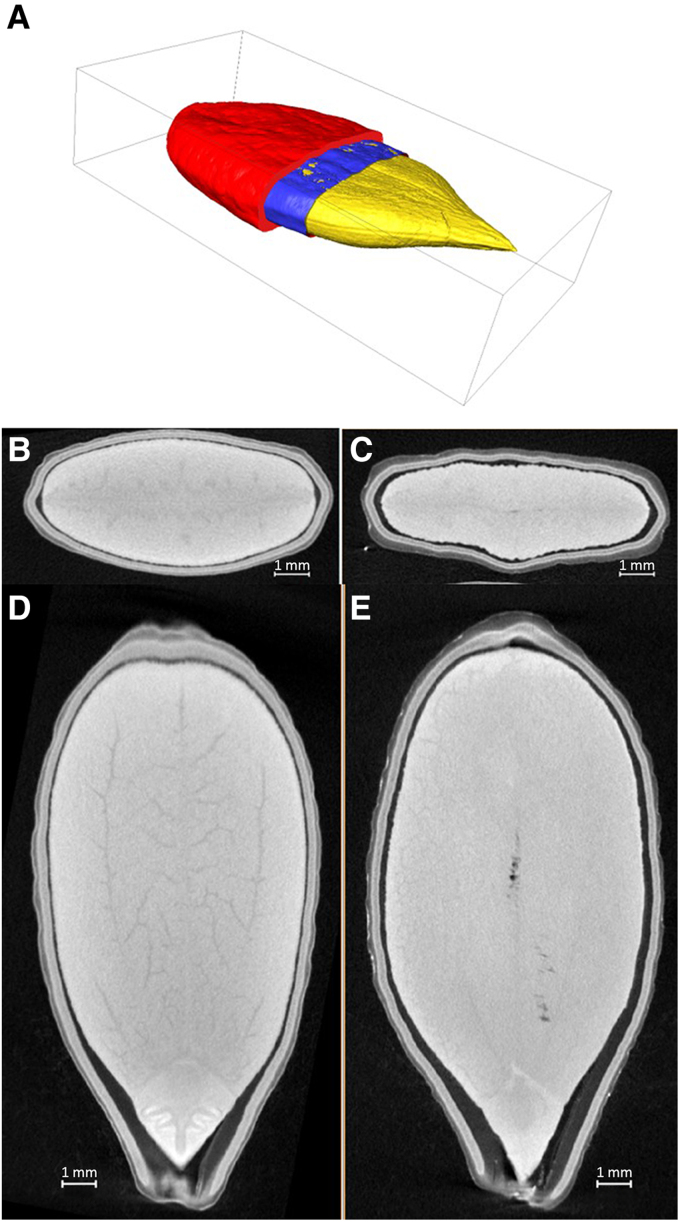
Micro-computed tomography (CT) of internal seed structure in wild cucumber. (A) Schematic representation of seed organization showing the three main components: internal tissues (yellow), air cavities (blue), and seed coat (red). (B, C) Longitudinal micro-CT sections of non-buoyant (B) and buoyant (C) seeds. (D, E) Transverse micro-CT sections of non-buoyant (D) and buoyant (E) seeds.

Micro-CT imaging provided detailed insights into the internal structure of buoyant and non-buoyant seeds, revealing key differences in tissue organization and air cavity distribution (Fig. 2). Buoyant seeds contained visibly larger and more continuous internal air spaces and had a thicker seed coat than non-buoyant seeds. In contrast, non-buoyant seeds showed denser internal tissue with only small, fragmented cavities and a generally higher total tissue volume. Quantitative measurements confirmed these trends: buoyant seeds had greater seed coat volume, whereas non-buoyant seeds had higher values of internal tissue volume and tissue surface area. Detailed values for individual seeds are provided in Table 4.

**Table 4.** Micro-computed tomography analysis of wild cucumber seeds

	Volume (mm^3^)		
	Coat	Internal seed tissues	Air cavities
Buoyant seeds	113.8	165.4	25.6
	89.0	172.1	24.9
	78.6	167.0	18.6
Non-buoyant seeds	98.4	230.8	15.1
	77.8	163.2	11.2
	62.3	187.5	13.6

The table presents the volumes (mm³) of three structures—internal tissues, air cavities, and seed coat—in three buoyant and three non-buoyant seeds.

### Hydrodynamic forces of seeds

**Fig. 3.**
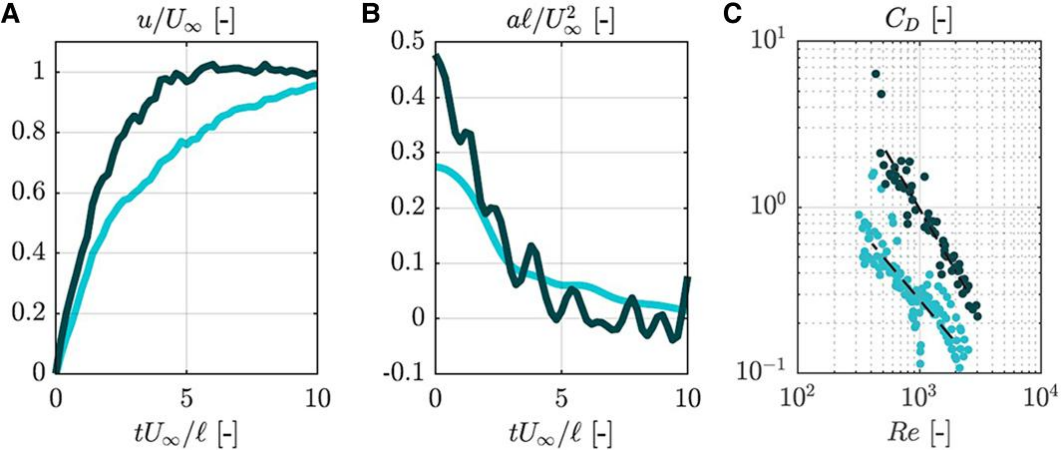
Velocity (A), acceleration (B), and drag evolution (C) in the channel experiment for buoyant (bright blue) and non-buoyant (dark blue) seeds. *tU_∞_/ℓ*=0 marks the seed release time. Dashed lines in the drag evolution plot designate power-law fits to the datapoints.

The results of the channel experiment, expressed in non-dimensional form using *U_∞_* and *ℓ* as characteristic velocity and length scales, are presented in Fig. 3. The streamwise velocity and acceleration were computed from trajectory data. Drag coefficients were plotted against Reynolds number (Re), showing distinct differences between buoyant and non-buoyant seeds.

The time to reach 95% of *U*_∞_ was 9.9*ℓ*/*U*_∞_ (0.48 s) for buoyant seeds and 3.9*ℓ*/*U*_∞_ (0.18 s) for non-buoyant ones. Peak acceleration was roughly twice as high for non-buoyant seeds. Drag coefficients followed:

*C*_D_≈110×Re^−0.866^ (buoyant), *C*_D_≈5820×Re^−1.258^ (non-buoyant)

with drag ratios between 2 and 5 depending on Re. For reference, a sphere at Re=1000 has *C*_D_≈0.44. Hence, buoyant seeds experience about half this value, and non-buoyant seeds nearly double.

**Fig. 4.**
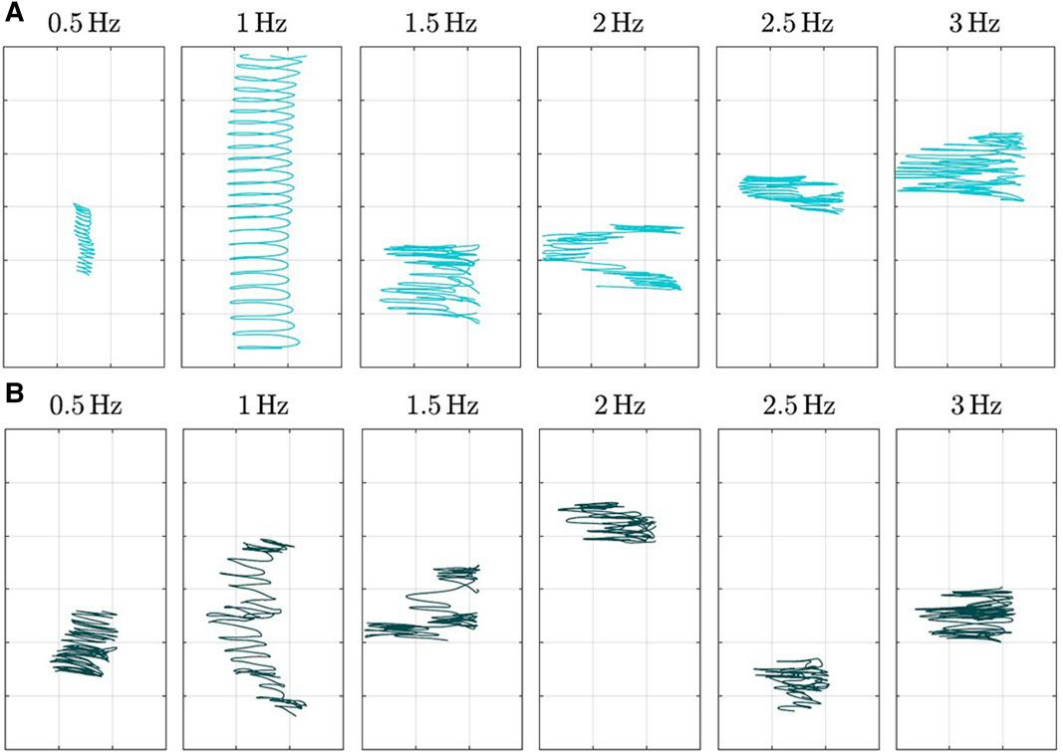
Sample trajectories of buoyant (A) and non-buoyant (B) seeds from the vessel experiment at different forcing frequencies *f*. Plot area is 120×60 mm.

At 1 Hz, buoyant seeds showed net linear translation perpendicular to table motion (i.e. along the *y*-axis), indicative of passive propulsion (Fig. 4; Video 3). This was combined with zero-mean rotations and bidirectional oscillations. Similar effects occurred at 0.5 or 1.5 Hz for some seeds. Non-buoyant seeds showed weaker and less consistent behavior.

**Fig. 5.**
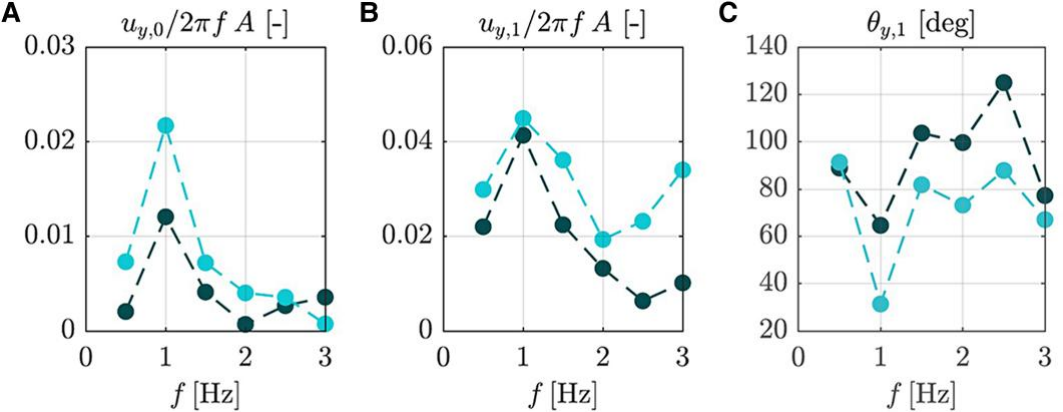
Parameters of the Fourier expansion of phase-averaged velocity along *y*-axis for buoyant (bright blue) and non-buoyant (dark blue) seeds at different frequencies in the vessel experiment. (A) Net translation velocity *u_y_*_,0_. (B) Oscillatory velocity component *u_y_*_,1_. (C) Phase lag θ*_y_*_,1_.

The net translational velocity component *u_y_*_,0_ exhibited a clear peak at 1 Hz for both buoyant and non-buoyant seeds, with the former achieving approximately twice the displacement of the latter (Fig. 5). The peak magnitude of *u_y_*_,0_ reached roughly 2.2% of the table’s maximum velocity, 2π*fA*. In contrast, the first harmonic component *u_y_*_,1_, which represents the zero-mean oscillatory motion, showed a much weaker dependence on the forcing frequency *f*. This indicates that while oscillatory motion persists across all frequencies, net translation is selectively amplified near 1 Hz. The phase lag θ*_y_*_,1_ remained close to 90° across most frequencies, implying that the seed acceleration is approximately in phase with the table’s velocity. Notably, a deviation from this trend occurred at 1 Hz, especially for buoyant seeds, suggesting enhanced correlation between seed and table velocities. This resonance-like alignment likely underlies the emergence of net translation at this specific frequency.

## Discussion

Our findings demonstrate that functional divergence in seed anatomy and hydrodynamic behavior underpins a dual dispersal strategy in *Echinocystis lobata*, providing a clear mechanistic basis for adaptive bet-hedging in dynamic riparian environments. Biometric analysis (Tables 2, 3) revealed significant seasonal variation in morphology, with spring-collected seeds generally larger and heavier—likely a result of prolonged maturation within fruit capsules and/or variable developmental environments (Baskin and Baskin, 2000; Fenner, 2012). Freshly collected autumn seeds showed similar features to dry spring seeds. This supports the hypothesis that moisture content and hydration state influence perceived seed shape (Harper *et al*., 1970; Meyer *et al*., 2007; Erb *et al*., 2013). Contradictory findings between our results and those of Dylewski *et al*. (2018), who observed that released seeds were larger than those remaining in the capsule, may stem from environmental variability, interannual differences, or even site-specific seed maturation processes. Such differences in seed size and mass are frequently influenced by factors such as resource allocation or the position of seeds within the fruit (Jakobsson and Eriksson, 2000; Khan *et al*., 2011; Dylewski *et al*., 2018). In our study, retained seeds were on average larger, but both retained and released seeds contained individuals capable of floating as well as sinking, indicating that buoyancy is not exclusive to either group and is likely influenced more by internal structure than by release timing. Approximately 20–30% of the seeds used in the experiment were buoyant, indicating that dispersal by water may play a role as a support mechanism in the invasion of this species. Additionally, observations suggested that freshly collected seeds exhibited a higher floating ability compared with dry seeds, implying that the potential for buoyancy may be greater than indicated by these results. However, this aspect was not deeply examined in the current study and warrants further investigation. The buoyant seeds did not differ significantly in mass from non-buoyant ones, but they exhibited a significantly larger surface area, resulting in a higher surface-to-mass ratio—a trait known to enhance buoyancy in many riparian species (Nilsson *et al*., 2010). Similarly, Carthey *et al*. (2016) found that seeds with a low volume-to-surface area ratio remained buoyant for longer, enhancing their potential for long-distance hydrochorous dispersal. These results resonate with the concept of functional traits facilitating hydrochory in riparian invasive plant species (Stromberg *et al*., 2008). In addition to single-seed transport, previous studies have documented that the entire dry fruit of wild cucumber can act as a dispersal unit and can be readily transported by running water (Bagi and Böszörményi, 2008). Fruits are lightweight, spiny capsules with substantially higher buoyancy than individual seeds, which may facilitate long-distance hydrochory, especially during high-flow events (Nilsson *et al*., 2010; Hyslop and Trowsdale, 2012). At the same time, buoyancy alone may not fully predict dispersal success in riparian habitats. Several studies have shown that many species without clear floating adaptations are present in waterborne seed banks and can be transported over considerable distances (Boedeltje *et al*., 2004; Nilsson *et al*., 2010). Other functional traits, such as fruit or seed shape, surface roughness, and the ability to trap air or adhere to drifting material, can contribute to buoyancy and affect the potential for water-mediated dispersal (Catford and Jansson, 2014; Carthey *et al*., 2016). Wild cucumber lacks specialized morphological adaptations for hydrochory (e.g. wings, persistent pappus) and appears to rely instead on the partial buoyancy of its diaspores and the action of hydrological events.

The DIC results (Fig. 1) indicate that moisture absorption induces structural changes in the seed coat, leading to localized deformations. The observed differences in swelling patterns may be related to the amount of available space within the seed coat, as well as the distribution of air cavities, as also observed in micro-CT imaging. Seeds with larger internal air cavities may experience more localized swelling in specific regions, whereas seeds with a more uniform internal structure might expand in a more even manner. The seed coat weakening and its control over water entry and dormancy release have been well documented in physically dormant species, including legumes (Collis-George and Melville, 1975; Souza and Marcos-Filho, 2001; Smýkal *et al*., 2014) Such structural traits may also interact with germination ecology. Non-buoyant seeds that sink and become incorporated into sediments are likely to persist in the soil seed bank, where burial can protect them from predation and desiccation and provide stable moisture for later germination (Leck and Brock, 2000). Autumn-released seeds require cold stratification to break dormancy and avoid premature germination before winter, a common adaptation among riparian annuals in temperate-continental climates (Baskin and Baskin, 2000). The observed variability in seed coat thickness and internal air cavities may therefore influence not only buoyancy but also water uptake and the timing of dormancy release, shaping both persistence and recruitment dynamics. DIC thus proves to be a valuable non-invasive method for visualizing the mechanical behavior of seeds during imbibition, especially in taxa where dormancy-breaking mechanisms are not yet fully understood.

The micro-CT imaging results (Fig. 2) show that the presence of internal air cavities in buoyant seeds likely contributes to their buoyancy, enhancing their ability to remain suspended in water for extended periods. Additionally, a thicker coat may restrict air escape and slow down the seed's water absorption. Similar traits have been noted in other species dispersed by water currents. For example, *Impatiens glandulifera* produces both buoyant and non-buoyant seeds, with the buoyant ones having larger air cavities and a thicker seed coat than the non-buoyant ones (Najberek *et al*., 2020). Catford and Jansson (2014) emphasized the role of traits like seed density, shape, and internal air space in determining water-mediated dispersal ability. This suggests that wild cucumber may employ a dual dispersal strategy, where both autumn-released and spring-released seeds contribute to population persistence and expansion. Viability tests confirmed that both buoyant and non-buoyant seeds are viable and capable of germination, supporting their potential to establish after dispersal. Seeds that fall immediately upon fruit dehiscence may remain close to the maternal plant, facilitating close-range generational turnover, which is particularly advantageous for short-lived annual species. In addition, seed release in *E. lobata* appears to be temporally staggered not only through variation in dispersal mode but also through physical retention. Approximately 34% of fruits retain viable seeds that are not immediately released at dehiscence (Dylewski *et al*., 2018). Such dual timing of dispersal potentially ensures both local regeneration and broader colonization during hydrological events. This type of within-species variation in buoyancy is not unique to wild cucumber. Similar variation in seed buoyancy has been observed in other species. For instance, *Sparganium emersum* produces both long-floating and short-floating seeds, and certain Amazonian riparian plants generate a mix of buoyant and sinking seeds, supporting diversified hydrochorous dispersal strategies (Williamson *et al*., 1999; Pollux *et al*., 2009).

Our results reveal two different hydrodynamic strategies (Fig. 3) between buoyant and non-buoyant seeds, reflecting distinct ecological adaptations. Buoyant seeds are characterized by lower drag, making them less responsive to high-frequency water motions and thus supporting long-distance dispersal (Murray, 2012). By floating in a stable, horizontal posture, they present minimal surface area to the current, minimizing drag and facilitating extended downstream movement (Murray, 2012; Carthey *et al*., 2016). In contrast, non-buoyant seeds accelerate rapidly and often orient perpendicularly to flow, increasing drag and promoting quicker sedimentation and localized deposition, consistent with observations in species such as *Hymenocallis coronaria* (Markwith and Leigh, 2008). Such dynamics suggest that buoyant seeds are more likely to exploit surface currents during high-flow events for long-distance dispersal, while non-buoyant seeds favor local recruitment. This dispersal duality has been documented in other species like *Impatiens glandulifera* or *Mimulus guttatus*, where this strategy directly influences colonization patterns (Truscott *et al*., 2006; Najberek *et al*., 2020). Importantly, the observed passive propulsion at ∼1 Hz oscillations (Figs 4, 5), more pronounced in buoyant seeds, points to a mechanism by which seeds may harness ambient flow oscillations to enhance transport. This phenomenon resembles mechanisms described in biomimetic systems that use surface waves to achieve directional motion without active propulsion (Rhee *et al*., 2022). Similarly, theoretical work in fluid dynamics has shown that passive bodies can achieve net movement in oscillating flow fields (Beal *et al*., 2006; Benham *et al*., 2024). These findings underscore the importance of seed morphology and flow regime interactions in shaping dispersal efficiency, highlighting hydrochory as a key driver of riparian plant community dynamics and invasive species spread (Säumel and Kowarik, 2013; Catford and Jansson, 2014). Incorporating such detailed hydrodynamic parameters into dispersal models can improve predictions of seed transport and deposition, thus informing conservation and restoration efforts in coastal ecosystems.

Dual strategy in buoyancy suggests a functional adaptation to varying dispersal environments and hydrological conditions (Beerling and Perrins, 1993; Brändel, 2004). Moreover, retaining part of the seed within the fruit may act as a passive defense mechanism against seed predation, as trapped seeds are less likely to be removed by rodents (Dylewski *et al*., 2018). However, seeds that remain buoyant for long periods and are deposited in the drift line can also be more vulnerable to predation and fungal or microbial attack, as this exposed zone is frequently colonized by rodents and pathogens (Fenner and Thompson, 2005). In such cases, floating dispersal may represent an ecological sink rather than a successful colonization pathway (Danvind and Nilsson, 1997; Boedeltje *et al*., 2004). Conversely, seeds with low or transient buoyancy that sink earlier and become incorporated into sediments may escape these risks and find more favorable conditions for germination (Leck and Brock, 2000). This temporal and functional variability supports the concept of dispersal bet-hedging, where seeds are split across time and ecological opportunities to maximize reproductive success in unpredictable environments (Childs *et al*., 2010; Tielbörger *et al*., 2012; Pausas *et al*., 2022). This may be particularly beneficial in riparian habitats subject to episodic flooding and disturbance. The presence of multiple dispersal vectors, including hydrochory and zoochory, further illustrates the ecological flexibility of wild cucumber, potentially enhancing its colonization capacity (Bagi and Böszörményi, 2008; Dylewski *et al*., 2019). Moreover, such a strategy aligns with broader patterns observed in riparian and invasive plants, where mixed dispersal modes (i.e. local retention and long-distance transport) enhance both colonization of new habitats and resilience of existing populations (Beerling and Perrins, 1993; Gallego-Fernández *et al*., 2021; Brändel, 2004). Ecological models have shown that splitting seed between early and delayed germination, or between high and low dispersibility, can maximize success under fluctuating conditions—this has been demonstrated in species such as *Blepharis sindica* and desert annuals (Venable and Lawlor, 1980; Narita, 1998).

**Fig. 6.**
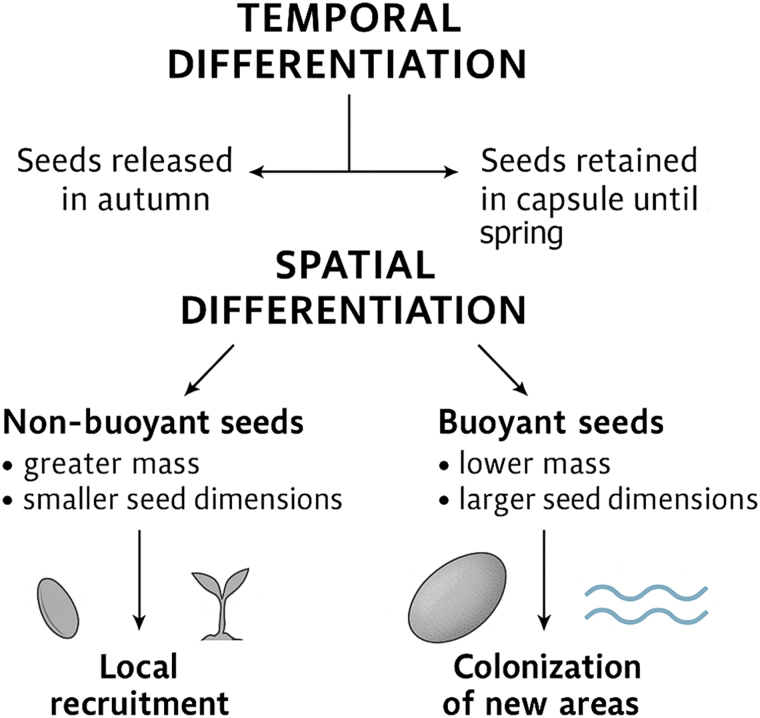
Conceptual model of seed dispersal strategies in wild cucumber. The diagram illustrates two interacting strategies of dispersal: temporal differentiation (autumn and spring released seeds) and spatial differentiation based on buoyancy (buoyant and non-buoyant seeds).

Synthesizing these findings, we propose a conceptual model of seed dispersal in *Echinocystis lobata*, structured along two main axes: temporal release (early and delayed) and buoyancy-mediated spatial mobility (buoyant and non-buoyant). Wild cucumber staggers seed release over time, with some seeds dispersed in autumn and others remaining within the fruit capsules until spring. This temporal variation may enhance survival under fluctuating environmental conditions and reduce predation risk, particularly for seeds retained over winter. In temperate-continental regions where wild cucumber occurs, both in its native North American range and in much of Central and Eastern Europe, winter snow cover followed by spring snowmelt often generates major flooding events that act as key dispersal pulses for riparian plants (Nilsson *et al*., 2013). Releasing part of the seed in spring appears particularly adaptive to this hydrological regime, as it allows buoyant diaspores to enter high-flow waters during peak flood events, maximizing downstream transport and colonization potential (Merritt and Wohl, 2002). Importantly, seeds dispersed in both autumn and spring show potential for buoyancy, enabling a spatial diversification of dispersal strategies. Non-buoyant seeds are more likely to remain near the maternal plant, promoting local recruitment and population persistence, whereas buoyant seeds can undergo hydrochorous transport, facilitating the colonization of new habitats (Fig. 6). This model aligns with broader concepts of evolutionarily stable strategies in annual species, where combining different timings and spatial dispersal modes reduces reproductive risk under unpredictable disturbance regimes (Venable and Lawlor, 1980; Childs *et al*., 2010). It highlights the significance of intraspecific variability in dispersal-related traits as a key factor contributing to invasiveness. The proposed framework offers a mechanistic explanation for how wild cucumber balances local persistence with range expansion and may provide a useful reference point for exploring dispersal strategies in other invasive species.

From a management perspective, these insights into the dual temporal–spatial dispersal strategy of wild cucumber can inform more effective control actions along invaded river ecosystems. Existing management recommendations for wild cucumber primarily focus on pulling or mowing entire plants before they produce seeds and on removing young seedlings in the spring (Koniakin *et al*., 2024). Our findings suggest that removing whole fruits in late summer or early autumn, before capsule dehiscence, could markedly reduce autumn seed rain and the seeds retained until spring. Because seeds require cold stratification, follow-up removal of spring seedlings remains essential to deplete the seed bank. For buoyant propagules, floating barriers or seed traps could help intercept diaspores during high-flow events. Although these devices have mainly been used for studying hydrochorous dispersal (Stevenson and Vargas, 2008; Londe *et al*., 2017), their demonstrated ability to capture drifting seeds suggests potential for reducing downstream spread in management applications. Prioritizing upstream populations and highly connected river sections would further reduce colonization pressure downstream.

## Supplementary Material

eraf526_Supplementary_Data

## Data Availability

Data supporting this study have been deposited in the Zenodo repository (Dołkin-Lewko and Baj, 2025; https://doi.org/10.5281/zenodo.17313254).

## Supplementary data

The following supplementary data are available at *JXB* online.

Fig. S1. Photographic documentation of buoyant and non-buoyant seeds before cold treatment and after 14 d of stratification followed by germination under controlled conditions.

Fig. S2. TTC viability test of buoyant seeds

Fig. S3. TTC viability test of non-buoyant seeds
