# Supplementary material for: Wild cucumber invasiveness: impact of seasonal changes on biometric seed traits and dispersal strategy
Source: J Exp Bot. 2025 Dec 4;77(6):1874–87. doi: 10.1093/jxb/eraf526 (PMC13103663; doi:10.1093/jxb/eraf526)
Supplement: eraf526_Supplementary_Data [file eraf526_Supplementary_Data.pdf]

Supplementary Material 1— Seed viability and germination tests

This supplementary file provides additional data supporting the ecological interpretation of the dual seed dispersal strategy in wild cucumber (*Echinocystis lobata)*. It includes photographic documentation of seed viability and germination tests that complement the main text. These additional materials allow readers to verify and better understand the viability and germination potential of both buoyant and non-buoyant seeds discussed in the article.

The preliminary germination test showed that both buoyant and non-buoyant seeds were able to germinate after cold stratification. Out of the 10 seeds tested in each group, eight floating and seven sinking seeds germinated (Fig. S1). To verify seed viability on a larger sample, a tetrazolium chloride (TTC) viability test was performed on 50 seeds from each group. In all cases the embryo axis stained red, indicating 100 % viability for both buoyant and non-buoyant seeds (Fig. S2, S3).

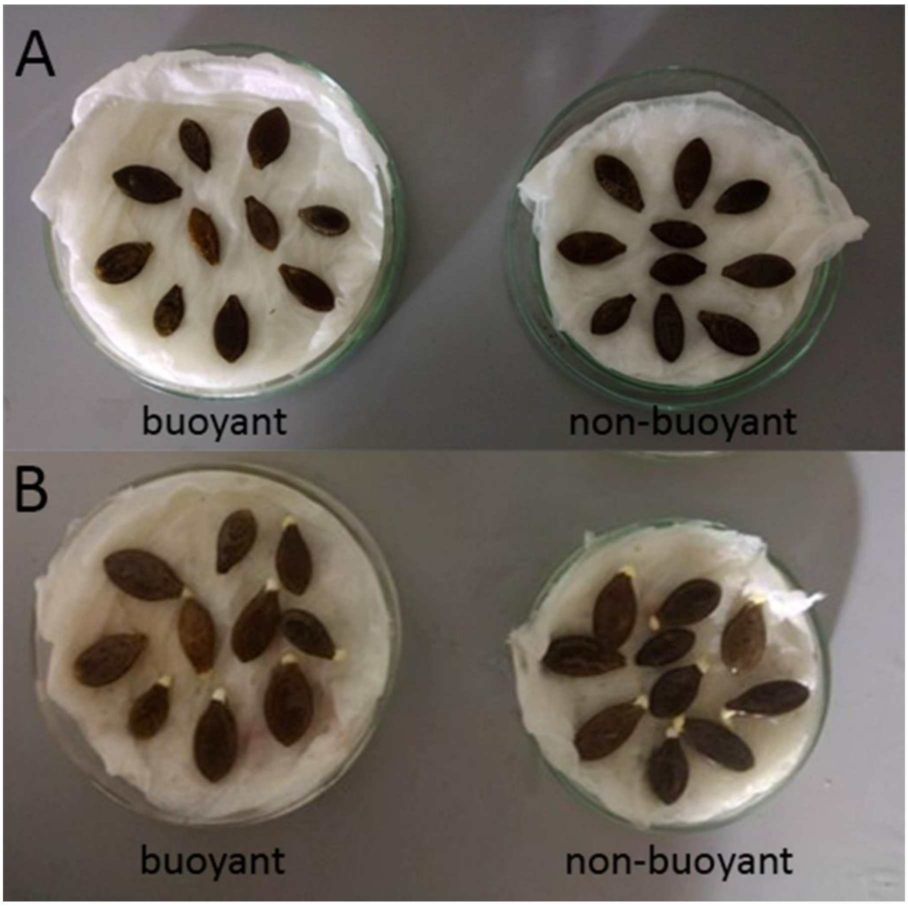

Fig. S1. (A) Buoyant and non-buoyant seeds at the beginning of the preliminary germination experiment, before cold treatment. (B) The same seeds after 14 days of cold stratification and subsequent germination under controlled conditions.

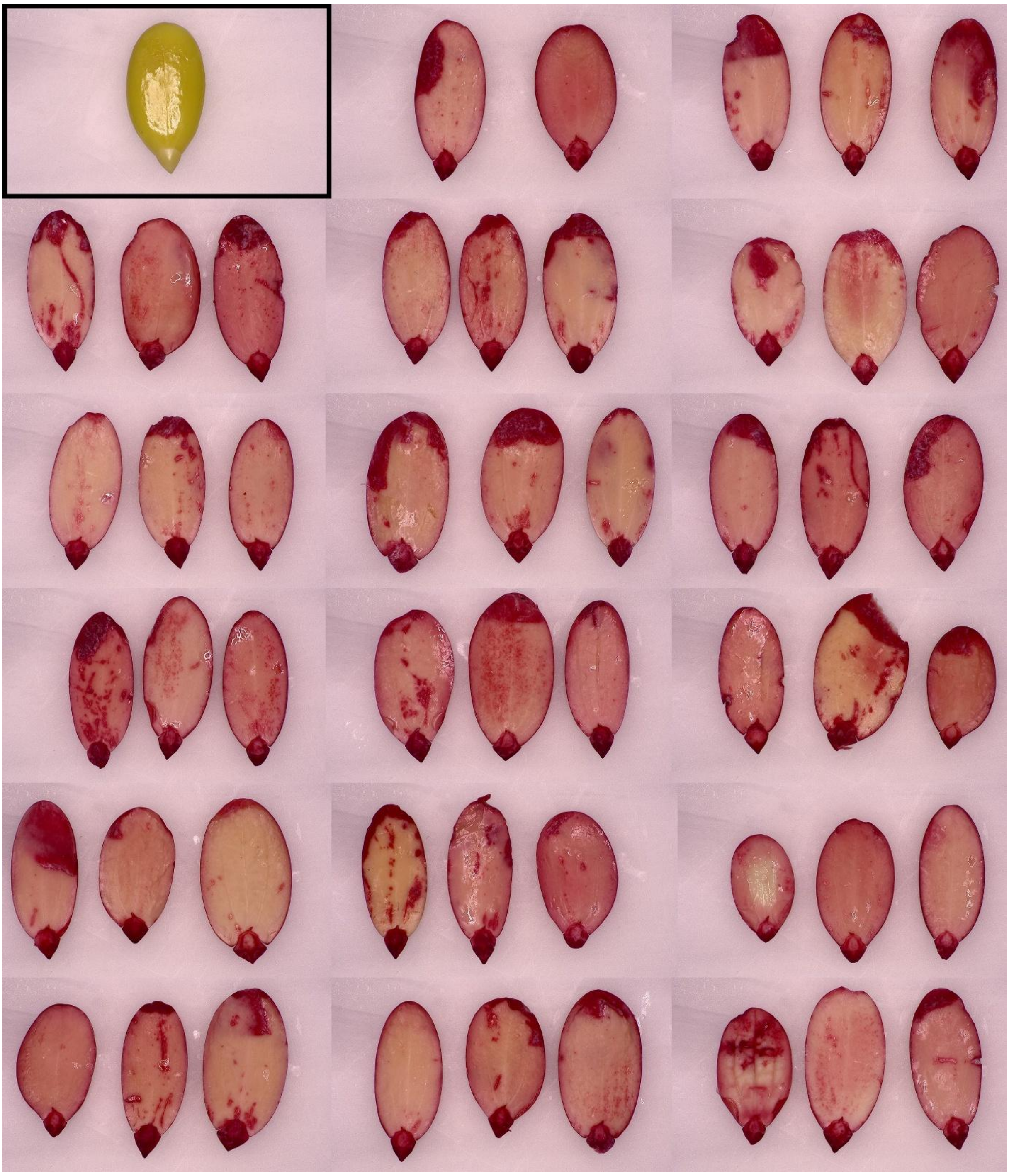

Fig. S2. Tetrazolium chloride (TTC) viability test of buoyant seeds. The seed shown in the black frame represents the negative control (boiled seed), which remained unstained, confirming that dead tissue does not take up the TTC dye. All other images show embryos from the tested sample (50 seeds) with a clearly red-stained embryo axis, indicating metabolic activity and viability.

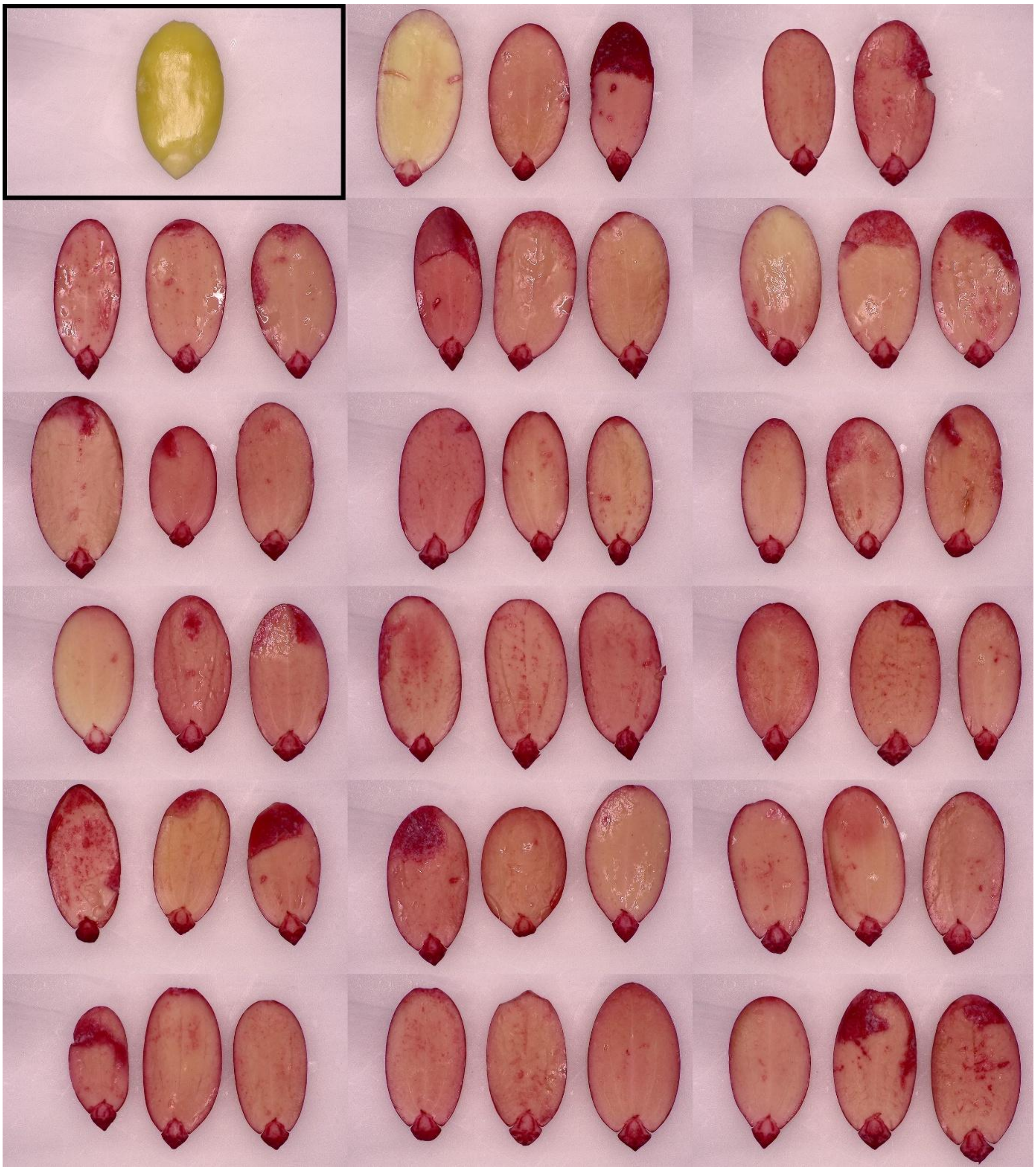

Fig. S3. Tetrazolium chloride (TTC) viability test of non-buoyant seeds. The seed shown in the black frame represents the negative control (boiled seed), which remained unstained, confirming that dead tissue does not take up the TTC dye. All other images show embryos from the tested sample (50 seeds) with a clearly red-stained embryo axis, indicating metabolic activity and viability.
